# Enterovirus-Induced Severe Rhabdomyolysis and Acute Fulminant Liver Failure in an Immunocompetent Adult Requiring Liver Transplantation: A Case Report

**DOI:** 10.7759/cureus.24336

**Published:** 2022-04-21

**Authors:** Santhalakshmi Angappan, Abdul Kader Tabbara, Jacob Pawloski, Anoop K Chhina, Dragos Galusca

**Affiliations:** 1 Anesthesiology/Critical Care, Perioperative Medicine and Pain Management, Henry Ford Health System, Detroit, USA; 2 Anesthesiology/Critical Care, Henry Ford Health System, Detroit, USA; 3 Neurological Surgery, Henry Ford Health System, Detroit, USA

**Keywords:** acute fulminant liver failure, immunoglobulin, liver transplant, rhabdomyolysis, enterovirus

## Abstract

The authors report a case of a young healthy adult with severe rhabdomyolysis and acute fulminant liver failure with multiple organ dysfunction syndromes (MODS), possibly from an enterovirus infection. To the best of our knowledge, this is the first-ever reported case of enterovirus-induced rhabdomyolysis and acute liver failure (ALF) in an immunocompetent adult. It is vital that the treating physician be aware of the association between viral infections, viral myositis, and severe rhabdomyolysis with acute liver failure, which can facilitate the optimal management of such patients. Prompt recognition may provide an opportunity for early interventions, including intravenous immunoglobulin and liver transplantation, if warranted.

## Introduction

Enteroviruses account for more than 10-15 million symptomatic cases per year in the United States [1]. The clinical presentation of human enterovirus infection can range from mild febrile illness to severe illness, including myocarditis, meningoencephalitis, and more rarely, acute liver failure (ALF) with a fulminant course, especially in pediatric and immunocompromised populations [2]. Most infections are self-limiting and require only symptomatic treatment. Enterovirus sepsis and severe hepatitis are more frequently seen in neonates [3], although there have been a few case reports of severe hepatitis in immunocompromised adults [4,5]. In addition, there have also been rare instances of successful liver transplantation in neonates as a treatment for acute liver failure due to disseminated enterovirus infection [6,7]. However, no similar association between enterovirus and rhabdomyolysis with acute liver failure has been described in immunocompetent adults.

Severe rhabdomyolysis from viral myositis is most often associated with mild elevations in liver enzymes. However, in rare circumstances, severe rhabdomyolysis can be associated with ALF, leading to multi-organ dysfunction in an immunocompetent adult. The authors report a case of a young healthy adult with severe rhabdomyolysis and acute fulminant liver failure with multiple organ dysfunction syndromes (MODS), possibly from an enterovirus infection. To the best of our knowledge, there has been no reported literature on enterovirus-induced ALF and severe rhabdomyolysis in immunocompetent adults. Informed written consent was obtained from the patient’s family for publication.

This article was previously presented as a meeting abstract at the 2020 SCCM Society of Critical Care Medicine conference in January 2021.

## Case presentation

A 25-year-old male from the United Kingdom, with no known co-morbidities and no previous history of illicit drug use, presented to an outside hospital in the United States (US) with a three-day history of flu-like symptoms. These symptoms included malaise, lethargy, and fever, which then progressed to altered mental status. History from family members revealed that the patient was not taking more than the prescribed dose of acetaminophen. The patient traveled to the US for a vacation. The patient's history was notable for a syncopal episode after a half marathon, 10 days prior to the travel, and herbal supplement (Thermopure) intake for bodybuilding. On evaluation at the outside hospital, the laboratory work was remarkable, as shown in Table 1.

**Table 1:** Laboratory values at presentation

Laboratory parameters	Patient values	Reference range
AST aspartate aminotransferase U/L	7500	10–40
ALT alanine aminotransferase U/L	7200	7–55
Total bilirubin mg/dl	8.5	0.1–1.2
Creatinine phosphokinase U/L	90,326	39–308
Ammonia mcg/dl	127	15–45
Creatinine mg/dl	4.8	0.7–1.35
Potassium meq/l	8.5	3.5–4.5
Lactate mmol/l	9.3	0.5–2.2

The patient was started on an N-acetylcysteine (NAC) infusion and hemodialysis and transferred to our hospital for escalation of care. On arrival, the patient was encephalopathic and in severe respiratory distress. His vitals were: a heart rate of 110/minute, blood pressure of 100/54 mm Hg, and respiratory rate of 32/minute. He was intubated for airway protection and tachypnea. Lab values were significant, as shown in Table 2.

**Table 2:** Laboratory values

Laboratory parameters	Patient values	Reference range
AST aspartate aminotransferase U/L	7777	10–40
ALT alanine aminotransferase U/L	6039	7–55
Total bilirubin mg/dl	7.6	0.1–1.2
Creatinine phosphokinase U/L	112,409	39–308
Ammonia mcg/dl	142	15–45
Creatinine mg/dl	3	0.7–1.35
INR international normalized ratio	9.5	0.8–1.1
Lactate mmol/l	5	0.5–2.2

**Figure 1:**
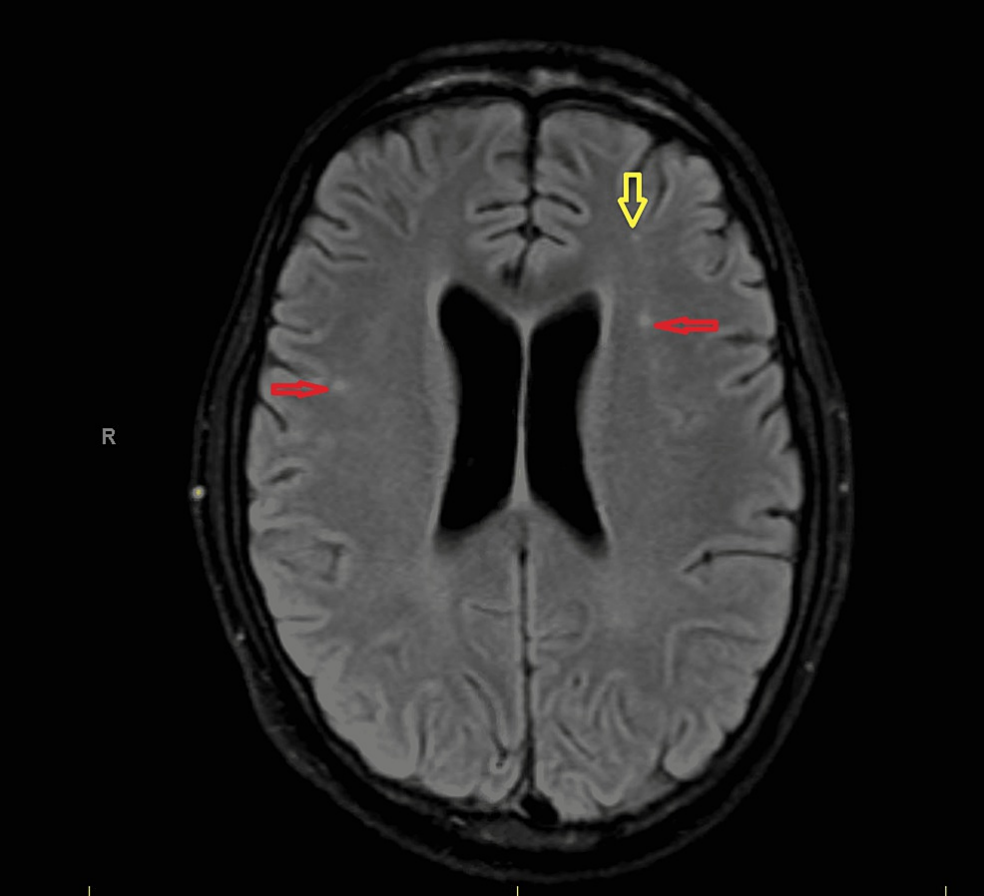
MRI brain without contrast: red arrows point to the small areas of high signal intensity on the FLAIR in the along the centrum semiovale; yellow arrows point to the high signal intensity on the FLAIR in the periventricular and subcortical white matter.

A liver transplant workup was initiated considering his hepatic encephalopathy and coagulopathy. He was started on empirical antibiotics, coagulation correction, and sustained low-efficiency dialysis (SLED). Workup for all infectious causes, both bacterial and viral, was negative except for bio-fire from the nasopharyngeal swab, which was positive for Enterovirus. Both the urine and stool polymerase chain reaction (PCR) for the enterovirus were negative. T2, urine legionella, Streptococcus pneumoniae, Epstein Barr virus (EBV) DNA, hepatitis serology, and (human immunodeficiency virus) HIV were negative. Eventually, the patient required an orthotopic liver transplant within the first 48 hours of presentation with a MELD score of 40. The explant liver pathology revealed microvesicular steatosis and coagulative necrosis. His postoperative course was complicated by persistently elevated CPK and severe ongoing rhabdomyolysis. The patient had to undergo multiple fasciotomies for bilateral lower extremity compartment syndrome. In view of his persistent rhabdomyolysis, he had a muscle biopsy which revealed necrosis without viral inclusion particles. The patient had a lumbar puncture which was negative for infectious etiology. The patient also had an MRI imaging of his brain (Figure 1), in view of his persisting altered sensorium. Imaging revealed multiple small vessel ischemic changes, not correlating with his age.

His liver function was returning to normal limits with immunosuppression on board. After a multidisciplinary discussion, the patient was started on intravenous immunoglobulin for high suspicion of enterovirus-induced. Following this, the patient’s clinical course improved significantly. The patient was transitioned from SLED to intermittent hemodialysis. The patient also had a tracheostomy and a PEG tube insertion in view of anticipated prolonged recovery. He was treated with antibiotics for ventilator-associated pneumonia (VAP). Over the course of time, his sensorium improved marginally, allowing him to obey simple commands and require minimal ventilator requirements. The patient continued to have persistent weakness and significant muscle wasting from the severe rhabdomyolysis.

In view of his anticipated prolonged recovery and need for prolonged rehabilitation, as per family request, he was transferred back to the UK in a hemodynamically stable condition. At discharge, the patient was on cyclosporine, mycophenolate mofetil, prednisone, and antibiotics for VAP coverage.

## Discussion

Enterovirus-induced severe rhabdomyolysis and acute liver failure were high on our differentials since the patient had flu-like symptoms before the presentation and tested positive (bio fire) only for enterovirus in his entire infectious workup. However, we were not able to isolate the virus in the stool or urine. Although the precise pathophysiology underlying virus-induced rhabdomyolysis is unknown, two mechanisms have been postulated: direct viral invasion and toxin-mediated injury [8]. There are reports where the virus cannot be isolated if the patient is out of the active infectious period and also the extensive muscle necrosis can be from the toxin-mediated injury from the viremia [8-10]. Moreover, viral particles are occasionally difficult to differentiate from glycogen by electron microscopy; therefore, there is some doubt about these observations. Biopsies of clinically affected musculature that are essentially unremarkable raise the possibility of a circulating "toxin" or cytokine causing rhabdomyolysis. Moreover, the patient showed significant clinical improvement after he was started on intravenous immunoglobulin, which favors enterovirus-induced illness. Though there are no case reports to date on enterovirus-induced severe rhabdomyolysis and acute liver failure in immunocompetent adults, there are case reports on enterovirus-induced rhabdomyolysis and acute liver failure in pediatric patients and immunocompromised adults. The patient was deemed a liver transplant candidate as per the Kings College criteria since his INR was greater than 6.5 and he had a non-hepatitis etiology for liver failure.

Acute liver failure is the deterioration of liver function that occurs rapidly in days or weeks, usually in a person who has no preexisting liver disease. ALF is most commonly caused by the hepatitis virus or drugs such as acetaminophen. As per the history obtained from family members, the patient did not take more than the prescribed acetaminophen pills. The patient was not on any combination pills that contained acetaminophen, which moved acetaminophen-induced toxicity down the differential list. The patient had a negative hepatitis serology panel. Uncommon etiologies include the enterovirus family, certain pesticide injections, and ayurvedic or herbal supplement intake. Herbal supplements have been implicated as a major cause of liver injuries. The herbal medications most commonly associated with hepatotoxicity include ephedra, black cohosh, kava, etc. However, our research on the herbal supplement Thermopure, which contains green tea extract, did not reveal any associations with severe rhabdomyolysis or acute liver failure. Strenuous exercise has been implicated with rhabdomyolysis; however, these dramatic CPK values in literature have never been associated with marathon running or heavy workouts, and their association with acute liver failure has not been described in the literature. 

## Conclusions

The authors present this case here to highlight the possibility of viral myositis in severe rhabdomyolysis and fulminant liver failure. It is imperative that the treating physician be aware of the association between viral infections and severe rhabdomyolysis with acute liver failure, which facilitates the optimal management of such patients. Testing for human enterovirus PCR in both stool and urine should be performed in cases of ALF and severe rhabdomyolysis with uncertain etiology.
